# Mapping adolescent first births within three east African countries using data from Demographic and Health Surveys: exploring geospatial methods to inform policy

**DOI:** 10.1186/s12978-016-0205-1

**Published:** 2016-08-23

**Authors:** Sarah Neal, Corrine Ruktanonchai, Venkatraman Chandra-Mouli, Zoë Matthews, Andrew J. Tatem

**Affiliations:** 1 Department of Social Statistics and Demography, University of Southampton, Southampton, SO17 1BJ England; 2 WorldPop, Department of Geography and Environment, University of Southampton, Southampton, SO17 1BJ UK; 3 Adolescents and at-risk populations, Department of Reproductive Health and Research, World Health Organization, 20 Avenue Appia, 1211 Geneva 27, Switzerland; 4 Flowminder Foundation, 17177 Stockholm, Sweden

**Keywords:** Adolescent, Fertility, Inequality, Spatial analysis, Small area estimation

## Abstract

**Background:**

Early adolescent pregnancy presents a major barrier to the health and wellbeing of young women and their children. Previous studies suggest geographic heterogeneity in adolescent births, with clear “hot spots” experiencing very high prevalence of teenage pregnancy. As the reduction of adolescent pregnancy is a priority in many countries, further detailed information of the geographical areas where they most commonly occur is of value to national and district level policy makers. The aim of this study is to develop a comprehensive assessment of the geographical distribution of adolescent first births in Uganda, Kenya and Tanzania using Demographic and Household (DHS) data using descriptive, spatial analysis and spatial modelling methods.

**Methods:**

The most recent Demographic and Health Surveys (DHS) among women aged 20 to 29 in Tanzania, Kenya, and Uganda were utilised. Analyses were carried out on first births occurring before the age of 20 years, but were disaggregated in to three age groups: <16, 16/17 and 18/19 years. In addition to basic descriptive choropleths, prevalence maps were created from the GPS-located cluster data utilising adaptive bandwidth kernel density estimates. To map adolescent first birth at district level with estimates of uncertainty, a Bayesian hierarchical regression modelling approach was used, employing the Integrated Nested Laplace Approximation (INLA) technique.

**Results:**

The findings show marked geographic heterogeneity among adolescent first births, particularly among those under 16 years. Disparities are greater in Kenya and Uganda than Tanzania. The INLA analysis which produces estimates from smaller areas suggest “pockets” of high prevalence of first births, with marked differences between neighbouring districts. Many of these high prevalence areas can be linked with underlying poverty.

**Conclusions:**

There is marked geographic heterogeneity in the prevalence of adolescent first births in East Africa, particularly in the youngest age groups. Geospatial techniques can identify these inequalities and provide policy-makers with the information needed to target areas of high prevalence and focus scarce resources where they are most needed.

## Background

Pregnancy in adolescence can present a major barrier to the health and wellbeing of young women and their children, and can contribute to long term educational and socio-economic disadvantage [1]. As we move towards the broader post 2015 Sustainable Development Goals agenda, attention is focussed more closely on how more nuanced indicators can highlight the needs of vulnerable sections of the population, and track how their needs are addressed through policies and programmes. National level targets may well be achieved by focussing on certain sectors of the population or geographical region whilst leaving other groups lagging behind, which again points to the need for demographic and spatial disaggregation to highlight disparities. A recent study by Neal et al. [2] in Uganda, Tanzania and Kenya highlighted the marked concentration of adolescent first births (particularly among younger adolescents) among the poorest and least educated sections of the population, and also found that progress over time was poorest amongst the most disadvantaged. The study also identified very marked geographic disparities in rates of adolescent first births within the three countries at state (administrative level 1) level.

Spatial inequalities in adolescent pregnancy and births are found in both high and low income countries, and are likely to reflect underlying levels of deprivation as well as inadequate access to reproductive health services [3]. In addition, adolescent motherhood is often strongly rooted in cultural practices, and this may well lead to prevalence (particularly among the youngest age groups) being concentrated within geographical “pockets” where communities share particular beliefs, norms and practices as well as possibly suffer high levels of deprivation. Spatial mapping of adolescent pregnancies has been used in developed country contexts to understand these geographic distributions, and has identified “hotspots” of adolescent births: small localities with high levels of adolescent childbearing [4, 5].

Prevalence mapping for disease or other adverse outcomes has become an important tool for policy makers in low income countries, and numerous studies have examined the spatial distribution of a range of maternal and child health and nutrition outcomes (e.g. [6–10]). While the value of this approach has been acknowledged with regards to adolescent programming (e.g. [11]) it has rarely been utilised for mapping the distribution of adolescent childbearing in low income country contexts. As the reduction of adolescent pregnancy is a priority in many countries, further detailed information of the geographical areas where they most commonly occur is of value to national and district level policy makers.

The aim of this study is to develop a comprehensive assessment of the geographical distribution of adolescent first births in Uganda, Kenya and Tanzania using Demographic and Household Surveys (DHS) data. In order to provide data that is useful for a range of policy makers and planners we present three separate approaches outlined by Ebener et al. [12] which can contribute to a greater understanding of spatial distribution of early first births:

- Descriptive/thematic mapping (creation of maps to convey information about a topic or theme) using choropleths
- Spatial analyses (extraction or creation of new information from spatial data) using adaptive bandwidth kernel density estimates
- Spatial modelling (spatial analysis that includes the use of statistical models to simulate phenomena) in a Bayesian framework.

These three approaches offer different perspectives and advantages for policy makers within the field of adolescent health. Descriptive mapping is generally used for presenting a visual representation of geographical variation for relatively large regions. It gives an overview of geographical inequities within countries, and a series of such maps can be used to highlight temporal trends or regions where progress in reducing adolescent births is particularly poor or good. Applying spatial analysis to information on adolescent childbearing using kernel density estimates provides an overall picture of “hotspots”, which is not constrained by administrative boundaries. This can be particularly useful when looking at correlations with other factors that transcend boundaries such as ethnic groupings. Finally, spatial modelling can be used to estimate rates of adolescent first births for small areas such as districts, using additional correlated variables. These are useful for identifying pockets of high prevalence, and can assist district level policy makers in setting priorities.

We present results from the application of these three approaches and thus produce an outline of the geography of adolescent childbearing in three countries disaggregated by age at under 16 years, 16–17 years and 18–19 years. Separating out the age groups enables births among the most vulnerable younger adolescents to be identified and mapped separately. Our discussion suggests how underlying factors may explain these geographic inequalities. It also outlines the advantages and disadvantages of the three different methods, as well as highlighting how policy makers have used such data in low income countries, and the potential for future use.

## Methods

### Data

Data were extracted for these analyses from the most recent Demographic and Health Surveys at the time of writing for Tanzania (2010), Kenya (2008), and Uganda (2011) [13–15]. The sample was restricted to women aged 20 to 29 at the time of the survey, resulting in sample sizes of *n* = 3347 Tanzanians, *n* = 3167 Kenyans, and *n* = 3284 Ugandans. Global Positioning Systems (GPS) coordinates of corresponding cluster locations were also gathered through the DHS and mapped using ArcGIS software version 10.2.2 [16]. Participant confidentiality is maintained by the DHS through cluster displacement of up to 2 km for urban clusters and 5 km for rural clusters. For these analyses, a total of 457 clusters were used for Tanzania, 397 clusters in Kenya, and 400 clusters in Uganda. Figure 5 in Appendix 1 shows the locations of the displaced clusters with associated sample size and urban/rural status. Of note, two districts in Tanzania contained no observed clusters containing women aged 20 to 29 years (Bukoba Urban and Pangani), while one district had only data for births between 18 and 19 years (Mafia). Data were weighted as outlined by DHS guidelines, using SAS version 9.4 software [17]. Administrative boundary shapefiles were obtained from the freely available Database of Global Administrative Areas (GADM) [18], while DHS regional shapefiles were obtained from the DHS [19], and projected using the World Geodetic System 1984 projection.

The outcome of interest was the percentage of women aged 20–29 at the time of survey who had given birth before the age of 20 years. As Neal et al.’s [2] earlier study found important differences in age patterns within the range of adolescent ages, we disaggregated the outcome into three different age groups: first birth before 16, 16–17 and 18–19 years.

### Descriptive mapping

Descriptive analyses were performed and presented in Table 1, by country and age group. In addition we produced descriptive choropleth maps using ArcGIS software version 10.2. These are thematic maps in which areas are shaded proportional to the measurement of the statistical variable being displayed: in this case age at first birth. As these descriptive maps are based directly on the survey estimates for the outcome it is not feasible to carry out analysis for small areas as small sample sizes result in large confidence intervals. Thus, the maps are presented at administrative level 1. These maps employed weighted outcomes, as outlined by DHS guidelines.

**Table 1:** Unweighted sample characteristics among female DHS respondents aged 20 to 29, by country and age at first birth (*N* = 9,798)

	Kenya (*N* = 3,167)	Tanzania (*N* = 3,347)	Uganda (*N* = 3,284)
	*N* (%)	*N* (%)	*N* (%)
DHS Survey year	2008	2010	2010
# of DHS Clusters	397	457	400
Any birth	2,403 (75.9 %)	2,641 (78.9 %)	2,742 (83.5 %)
*Mean age at first birth*	*19.1 ± 2.9*	*19.3 ± 2.6*	*18.7 ± 2.9*
Less than 16 years	324 (10.2 %)	212 (6.3 %)	435 (13.2 %)
*No education*	*105 (32.4 %)*	*85 (40.1 %)*	*69 (15.9 %)*
*Poorer or poorest quintiles*	*166 (51.2 %)*	*97 (45.8 %)*	*196 (45.1 %)*
16 to 17 years	546 (17.2 %)	633 (18.9 %)	700 (21.3 %)
*No education*	*87 (15.9 %)*	*190 (30.0 %)*	*110 (15.7 %)*
*Poorer or poorest quintiles*	*234 (42.9 %)*	*267 (42.2 %)*	*320 (45.7 %)*
18 to 19 years	676 (21.3 %)	855 (25.5 %)	766 (23.3 %)
*No education*	*99 (14.6 %)*	*174 (20.4 %)*	*95 (12.4 %)*
*Poorer or poorest quintiles*	*263 (38.9 %)*	*353 (41.3 %)*	*340 (44.4 %)*

### Spatial analyses

Kernel density estimation (KDE) is a non-parametric method for estimating density, and uses all the data points to create an estimate of how the density of events varies over a given area [20]. It produces a smooth map in which the density at every location reflects the number of points in the surrounding area. This can then be used to create prevalence surfaces, or heat maps, by generating a ratio of case data to control data. We used this method to create heat maps of adolescent first births with the prevR package in R software [21]. Further details of the methodology used can be found in Appendix 2, and are described elsewhere in the literature [22].

### Spatial modelling

The Integrated Nested Laplace Regression (INLA) modelling approach is a technique that can be used for small area estimation, which involves the estimation of parameters of sub-populations confined within a small geographical area as part of a larger survey population. It utilises a Bayesian hierarchical spatial regression modelling approach and was carried out here using the INLA package in R [23]. Such geoadditive models incorporating the INLA technique have been used previously in the DHS literature as a method to control for spatially correlated effects in a Bayesian framework [24]. By utilising a Bayesian framework, uncertainties in estimates can be quantified and presented, suggesting where future data collection efforts might be focussed.

For these analyses, proportions are presented at the administrative unit 2 level for Tanzania and Kenya, while the administrative unit 1 level was used for Uganda due to the high number of districts within the country (*n* = 168). By presenting provincial prevalence within Uganda, parity between geographical units can be maintained. Further methodological details can be found in Appendix 2, while associated confidence intervals and standard deviations for estimates are presented in Appendix 3.

## Results

### Sample characteristics

Overall, a total of 9,798 respondents were used in these analyses, utilising surveys administered between 2008 and 2010. Overall, 79.5 % of women (*N* = 7786) reported having any children by the time of survey, with mean age at first birth 19.0 years ± 2.8 years. Among this group of parous women, 9.9 % (*N* = 971) experienced first birth at less than 16 years old, while 19.2 % (*N* = 1879) had their first birth between ages 16 and 17, and 23.4 % (*N* = 2297) between ages 18 and 19. Among those having their first birth at less than 16 years of age, 26.7 % (*N* = 259) of women reported having no education and 47.3 % (*N* = 459) fell into the bottom two wealth quintiles, as defined by the DHS. Finally, after applying population-normalized DHS weights to ensure representation at the multi-country level, regional prevalence of first birth at less than 16 years was found to be 9.8 %, while prevalence of first birth between 16 and 17 years of age was 19.8 and 24.9 % between 18 and 19 years. Tables 1 and 2 show these sample characteristics broken down by country.

**Table 2:** Weighted prevalence of adolescent motherhood among female DHS respondents aged 20 to 29, by country

	Kenya (*N* = 3,167)	Tanzania (*N* = 3,347)	Uganda (*N* = 3,284)
	(%)	(%)	(%)
Less than 16 years	9.0 %	7.3 %	14.0 %
16 to 17 years	17.8 %	20.2 %	21.9 %
18 to 19 years	22.5 %	28.1 %	24.2 %

**Fig. 1:**
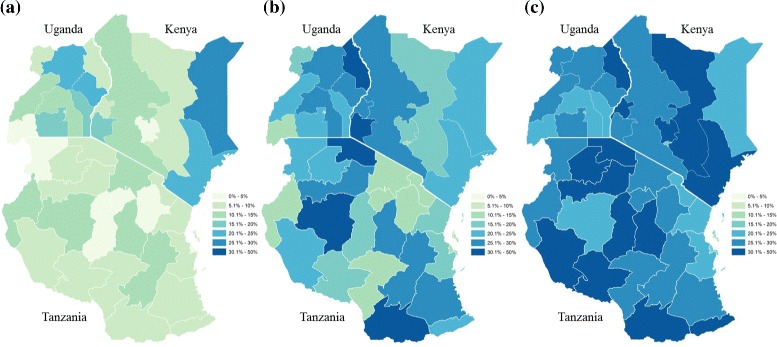
Weighted proportion of adolescent birth in East Africa among DHS respondents aged 20 to 29, at **a** less than 16 years old, **b** 16 to 17 years old, and **c** 18 to 19 years old

To provide a regional picture of adolescent first births in East Africa, choropleth maps were generated for DHS regions. Figure 1 reflects weighted sub-national proportions of women who had their first birth at less than 16 years old (Fig. 1a), from 16 to 17 years old (Fig. 1b), and 18 to 19 years old (Fig. 1c). Kenya and Uganda show marked geographic heterogeneity for first births under 16 years: for instance eastern Kenya and parts of Uganda have more than 20 % of women having a first birth before the age of 16, whereas for much of the country the figure is less than 10 %. In Tanzania there appears to be less geographical variation. Generally, as overall prevalence of first birth increases for ages 16/17 and 18/19 the heterogeneity also decreases.

### Spatial analyses

**Fig. 2:**
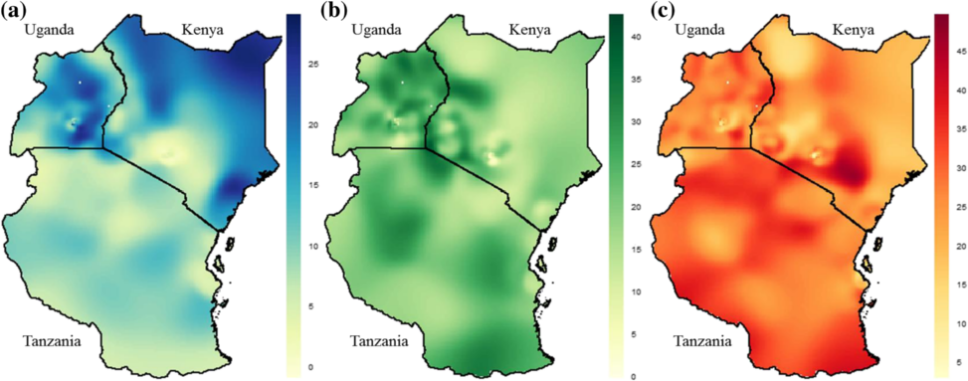
Regional heat map of adolescent birth in East Africa estimated by adaptive bandwidth KDE approach, at **a** less than 16 years old, **b** 16 to 17 years old, and **c** 18 to 19 years old

Prevalence surfaces, or “heat maps”, of maternal age at first birth were generated using an adaptive bandwidth technique encompassing an optimal number of persons surveyed through the DHS, similar to a nearest neighbour approach. The optimal N parameter (N_opt_) used in these analyses is defined in Appendix 1, and has been published in detail elsewhere [22]. Figure 2a represents the percentage of women having their first birth before 16 years old, while Fig. 2b and c represent ages 16 to 17, and 18 to 19 years respectively. Prevalence of childbearing tended to increase with increasing age; therefore, to emphasize within-group regional heterogeneity, varying scales were used between age categories, as specified in the corresponding legend key for each map. This was done to highlight areas within East Africa which might have high prevalence of birth in a given age category, even though this proportion might be lower as compared with other age categories. The kernel density maps broadly correlate with the choropleths, although the different scales bring out more clearly inequities in the 16/17 and 18/19 year age groups. Again, there is less variation in Tanzania for all age groups than for Kenya or Uganda. The lack of constraint from administrative boundaries allows us to see how “hot spots” or “cool spots” cross and are unaffected by country boundaries e.g. there is an area of lower prevalence that spread along the border between Uganda and Kenya for adolescent births <16 years, as well as several areas of higher prevalence than traverse the borders between Tanzania and Kenya for all three age groups.

### Spatial modelling

**Fig. 3:**
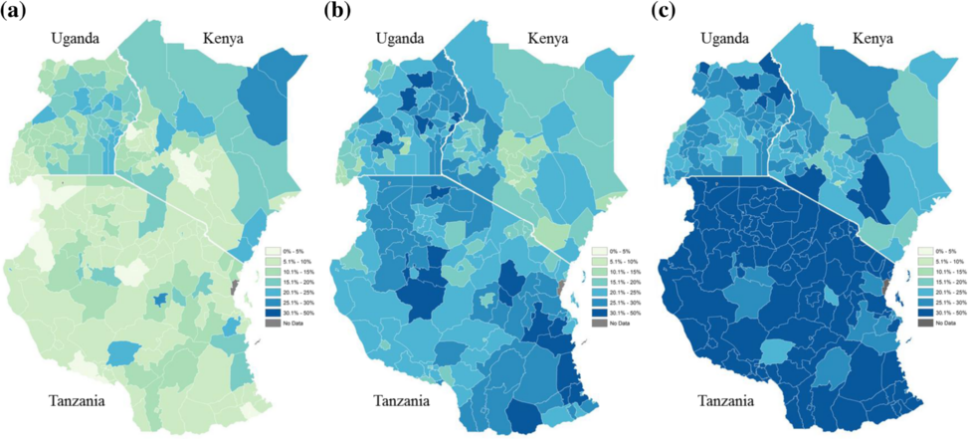
Predicted prevalence of adolescent birth in Kenya, Uganda and Tanzania estimated by Bayesian modelling, at **a** less than 16 years old, **b** 16 to 17 years old, and **c** 18 to 19 years old

Predicted prevalence of maternal age at first birth is shown in Fig. 3 for less than 16 years (Fig. 3a), between 16 and 17 years (Fig. 3b), and 18 to 19 years (Fig. 3c). To emphasize within-country variation in prevalence across age groups, countries are presented in columns by age categories for Fig. 3a–c. As would be expected there are strong similarities between the maps produced by the three techniques (and the choropleth and INLA for Uganda based on the same administrative unit level are highly comparable). However, for Kenya and Tanzania the INLA technique provides more nuances and detailed estimates as compared to the previously mentioned techniques. While it again broadly complies with both the choropleths and the kernel density maps, it suggests in some cases marked differences in neighbouring districts, which come out less clearly using the other two methods e.g. it highlights high levels of first births under 16 years in Mbarali district, Tanzania.

To reflect uncertainty in the mean estimates displayed in Fig. 3, we mapped standard deviations of the posterior distribution for each district, with corresponding 95 % confidence intervals listed in Appendix 3. These standard deviations reflect the range under which the presented estimates may fall, thereby providing an overall representation of variability within a given district which may assist policy makers in understanding the degree in which they can rely on the data for decision making. In general, the distribution of standard deviations approached normality with increasing age, most likely due to more frequent outcomes, or births (Fig. 6). Areas with highest associated standard deviations at less than 16 years of age included the north eastern region of Kenya and coastal areas of Tanzania. Such variation is likely a result of increasingly rare outcomes in more rural areas with already low sample sizes, and suggest future analyses examining adolescent motherhood might benefit from more focussed data collection efforts in rural areas. The area with highest standard deviation occurred in Mafia, an island off Tanzania’s coast, for births between 18 and 19 years. Most notably, births at less than 16 years old and between 16 and 17 years were not observed for this region, while births between 18 and 19 were also low, resulting in high standard deviation and wide confidence intervals (SD: 0.20; 97.5 % CI: 0.07–0.81). Detailed posterior distribution parameters for each region are outlined in Tables 3 through 5 in Appendix 3.

The disaggregation by age group for the three countries makes it possible to note emerging age-related patterns. In Tanzania very few districts have significant numbers of births under 16 years, but this is not the case for Kenya and Uganda. However, for the 16/17 age groups there are a number of districts with very high proportions of first births in all countries including Tanzania, as well as marked heterogeneity between regions. If we consider the 18/19 age group, there is less heterogeneity as most districts have high rates of first birth, although Kenya still has a number of regions with relatively low proportions. Most districts or regions show the pattern that would be expected where the proportion of first births increases with age, but there are exceptions: for example Mandera and Wajir actually have higher percentages of first births at <16 years, and these decline for 16/17 and 18/19 years (presumably because the majority or women have already given birth before these later age groups).

## Discussion

The findings show marked geographical heterogeneity for adolescent first births, particularly in Kenya and Uganda. The distribution in Tanzania, however, is more homogenous, at least for the <16 and 18/19 year group. These differences are most marked for the <16 age groups. While the INLA estimates at district level reflect broader patterns shown in the regional level choropleths, the more detailed maps are in some cases able to demonstrate “hot spots”, with marked heterogeneity across neighbouring districts.

A proportion of this heterogeneity is likely to reflect differences in underlying socio-economic determinants of adolescent fertility such as poverty and education. Previous work in Uganda and Kenya using small area estimation techniques has clearly demonstrated heterogeneity at the district level for various economic status indicators [25, 26], and indeed there is marked correlation of “hot spots” of poverty with our own estimates of high prevalence of early first births. Probably the most marked area of high prevalence are found in Kenya in the Mandera and Wajir region. While the standard errors are relatively large due to these regions being sparsely populated, the findings are plausible as they generally have very poor socio-economic indicators: they are ranked the second and third poorest districts in the country [27]. In addition this area is most populated by nomadic pastoralists, including many from the Somali ethnic group (some of whom have arrived as refugees from the conflict in Somalia). These populations have strong traditions of early marriage, as well as low levels of autonomy for women [28, 29]. In Uganda, the eastern districts with high levels of first births under 16 also have quite high levels of poverty, as well as having been affected by conflict, with a number of regions still experiencing high levels of displaced populations or food insecurity. Some findings are more difficult to explain. While moderate uncertainty parameters suggest the estimates should be interpreted with caution, some districts with very high levels of poverty in northern Uganda actually have relatively low levels of first births <16 years, which suggest cultural differences. Conversely, Mbarali district in Tanzania has a relatively high level of first birth <16 years compared to neighbouring districts, yet it is relatively wealthy. However, it does have a prevalence of HIV infection higher than the national average [30] which may suggest particular norms in sexual behaviour, and in addition to its geographical position on the Dar-Es Salaam – Mbeya corridor these findings may warrant further investigation. The area also has a large number of Maasai migrants who have a strong culture of early marriage, so this may also partially explain the findings [28, 29]. The apparent high rate of first births to women under the age of 16 years in Dodoma Urban also warrants further analysis. The lesser degree of geographical heterogeneity in Tanzania is difficult to conclusively explain, but may partly reflect the lower level of socio-economic inequity compared with Kenya and Uganda as measured by the Gini coefficient of inequality and the percentage inequality in income [31]. A further reason could be explained by differences in ethnic composition: the Tanzanian population is composed of a large number of smaller ethnic groups, which may mean diversity between ethnic groups is less clearly visible within a geographical context (or indeed there may be less ethnic diversity among the groups in terms of adolescent pregnancy).

Differences could at least partly reflect differing access to contraception: DHS reports show wide geographic variations in contraceptive prevalence in all three countries [13–15]. However, contraceptive uptake to prevent first births in nulliparous women is extremely low in all three countries (5 % in Tanzania and Uganda, and 14 % in Kenya [13–15]), so this is probably not a major factor.

The high levels of first births in young women under the age of 16 years in some parts of Kenya and Uganda is particularly concerning: there is evidence that the health disadvantages faced by both adolescent mothers and their infants are concentrated among younger adolescents, so should be of particular concern to policy makers [32–34]. The disaggregation by age groups allows us to ascertain age-related patterns which are often lost in studies that use a single indicator for adolescent births. Several areas such as Mandera and Wajir require further investigation and possible interventions, as do other districts in Uganda and Tanzania where rates appear high. High rates of first births at an early age suggest areas where appropriate services and information must be made available at a young age before sexual activity commences, which may require a markedly different approach to those targetted at older adolescents to allow for different levels of cognitive and emotional development. In addition further investigation is needed to understand the contexts of these pregnancies (e.g. within or outside marriage) to enable a comprehensive approach to addressing the issue [35]. In many contexts this will ensure developing and enforcing legal frameworks to establish age at marriage and protect girls from abuse and exploitation.

### Using mapping and Geographic Information System (GIS) techniques to inform policy and planning

This work provides examples of how mapping and spatial analyses using already-existing data can inform policy-makers about locations where the prevalence of adolescent pregnancies is high. In recent years there has been a marked increase in the number of studies drawing on geospatial techniques to either map health indicators or examine geographical access to services (e.g. [7–9]). The growing availability of georeferenced information available through large scale surveys such as the DHS provide further opportunities to use these methods in low and middle income countries to guide policy and practice. Using a variety of methods, as in this study, enables findings to be triangulated to confirm areas of potential concern. Such methods may need to be supported by more detailed analysis of local level data from either existing sources such as vital registration in areas where this data is available for the majority of the population, or health records where nearly all births occur within the health system. Alternatively, it may be necessary to gather focussed and specific data collection methods which can provide more nuanced information and assist in the development of strategies to respond to need.

When we specifically look at how geospatial data has been integrated within policy and planning for adolescent pregnancy prevention, the UK Teenage Pregnancy Reduction strategy developed in 1999 provides an interesting example [36–38]. This included the collation and dissemination of ward-level data on teen conceptions in order to identify “hotspots” (defined as more than 6 % of 15–19 year olds becoming pregnant). These high prevalence neighbourhoods could then be targeted in terms of resources and interventions.

**Fig. 4:**
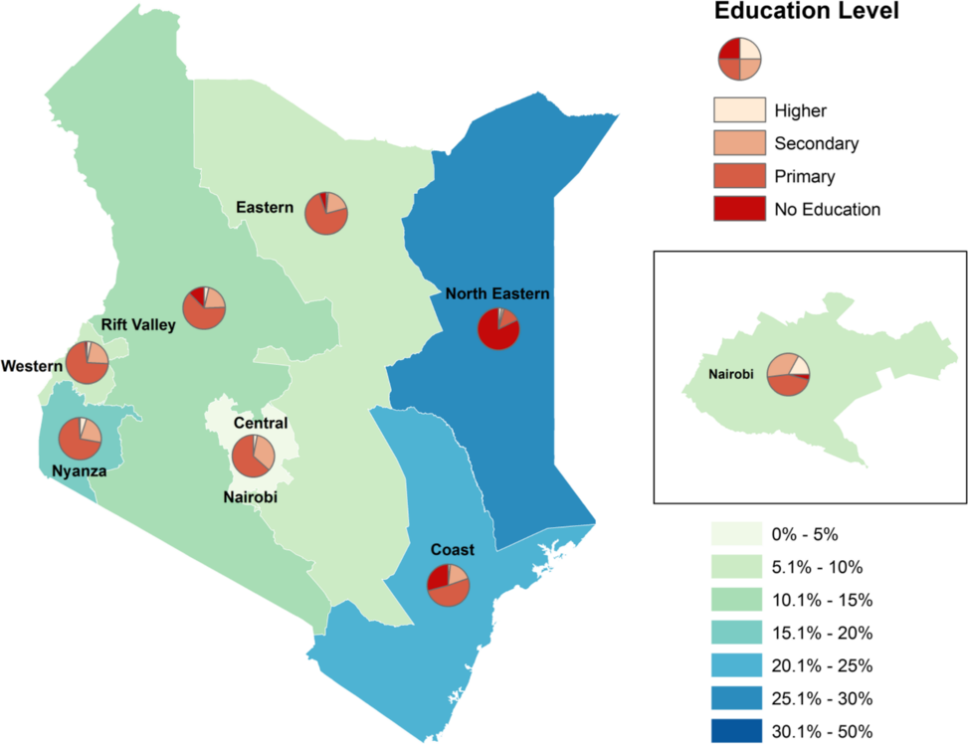
Weighted level of education and first birth at less than 16 years old by province, Kenya DHS 2008

**Fig. 5:**
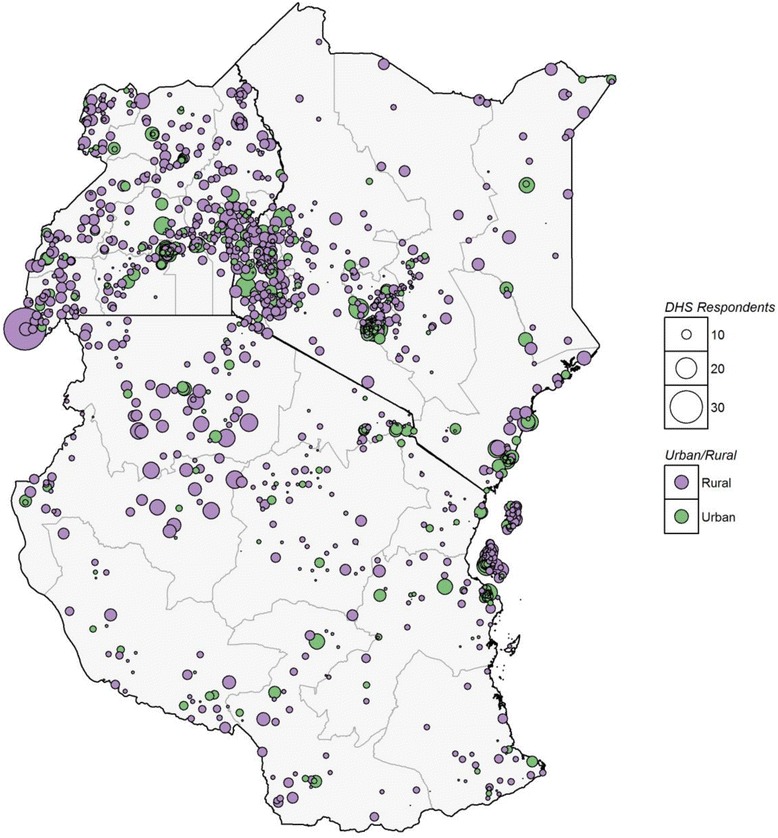
Displaced DHS cluster locations (*N* = 1254) with corresponding sample size and urban versus rural status

**Fig. 6:**
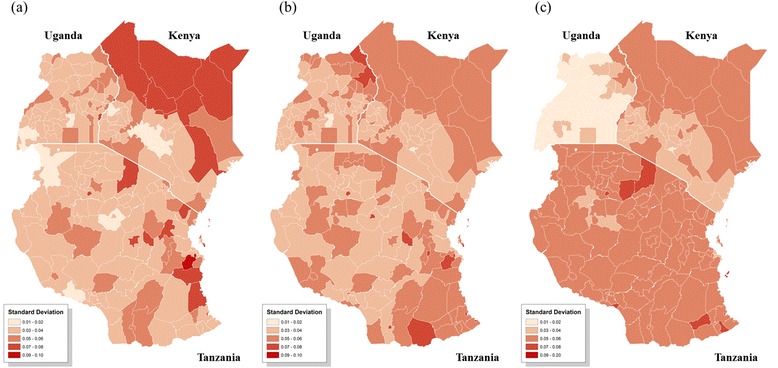
Standard deviations of predicted INLA estimates, at **a** less than 16 years old, **b** 16 to 17 years old, and **c** 18 to 19 years old

This paper has focussed on thematic mapping to identify areas of high adolescent prevalence. However further opportunities exist for using mapping and other geospatial modelling techniques to examine associations with other variables, or attempt to explain variance. At a simple level it is possible to layer different variables onto choropleth maps to show how different attributes may be associated to provide a clear visual representation: an example is shown in Fig. 4 which demonstrates the association between lack of education and births before age 16 years by region in Kenya. However, it must be noted that this is not always feasible at a small area level: the regional administrative level used for the map in Fig. 4 may restrict its value to policy makers. Alternative methodologies have been used to investigate how relationships between adolescent motherhood and underlying determinants vary spatially [4, 39] and this offers opportunities for further analysis in low and middle income countries.

### Limitations and advantages of geospatial techniques

The mapping techniques demonstrated in this paper have respective advantages and limitations for policy makers. The initial choropleths presented in this study based on prevalence are easy to carry out and present a visual representation of direct estimates that is easy to interpret. The technique does not need georeferenced data and can be created using free software. However, they cannot be used for small areas based on most national survey data sources as sample sizes will be too small and confidence intervals too great, which may limit their value to policy makers: this however may not be the case for data sources that do not rely on sampling (e.g. vital registration or census data). Kernel density estimates show “heat maps” of high prevalence areas that can be identified independently of administrative boundaries, and this can both be a positive or a negative attribute: when examining the relationship between adolescent motherhood and factors not affected by boundaries such as ethnicity it may be an advantage, but may be a disadvantage for policy makers keen to understand levels specifically within their own districts or regions. INLA can provide small area information which can be tailored to match the relevant administrative unit for health, thus making it particularly valuable for policy makers and theoretically at least can be used on very small areas, making it less likely that smaller hotspots are overlooked. It must however, be remembered that this is modelled data rather than direct estimates, and attention should be paid to estimates of uncertainty when interpreting the results. The uncertainty estimates for this study vary, but in some districts it suggests that results should be interpreted with caution, particularly where the estimates are wide, and supports the need for triangulation of data from other sources to guide programmatic decisions. While freely available via open source software, it requires fairly specialized knowledge or staff to implement in a programmatic setting. The use of a number of different techniques as included in this study offers an opportunity to triangulate findings and present more robust evidence.

There are also possible limitations associated with the use of DHS data, which relies on retrospective reporting of birth histories to identify adolescent births. This may be prone to either intentional or unintentional recall bias around age at first birth, and in particular there is some evidence that very young adolescent births may be under-reported: a previous study suggests this is most likely when using a sample of 15–19 year olds [40], so our use of a sample of 20–29 year old women should minimise this. Further potential bias may be introduced as the survey will record the birth at the place where the mother was residing at the time of the survey, not where she was at the time of the birth.

## Conclusion

Our studies demonstrate marked geographical heterogeneity in adolescent first births, particularly in Uganda and Kenya. These inequities are particularly marked for births under the age of 16 years, which is the group most likely to experience adverse outcomes from pregnancy for themselves and their infants The use of these three geospatial techniques enable these differences to be examined at regional and, in the case of Kenya and Tanzania, district level, as well as being able to display prevalence without the constraints of administrative boundaries. The use of several different methods allow results to be triangulated and enables greater confidence in the results. Such findings can provide policy-makers with the information needed to target areas of high prevalence and focus scarce resources where they are most needed. Geospatial methods have already proved valuable in guiding policy in developed countries and the proliferation of georeferenced data through surveys in low income countries offers greater opportunities to understand and address geographic inequities.

## Abbreviations

DHS, Demographic and Household Surveys; GIS, geographic information systems; INLA, Integrated Nested Laplace Approximation; KDE, Kernel density estimation

## Appendix 2: Further details of methodology used for Kernel Density Estimation and INLA

### Kernel density

Because DHS data uniquely employ clusters comprised of both cases and controls, spatial independence cannot be assumed when using these data. To address this limitation, the prevR package was developed by Larmarange et al. [22] specifically for use with DHS data to create smoothed prevalence surfaces using adaptive bandwidth kernel estimator techniques, with radii width varying to encompass an equal number of persons surveyed. This technique is advantageous over other fixed and adaptive bandwidth kernel estimator approaches specifically in regards to DHS data, where cases and controls for a given system cannot be assumed to be spatially independent of each other, and often are in fact geographically located within the same DHS cluster.

To create a unified prevalence surface of the East Africa region, DHS data from all three countries were pooled prior to analysis. Of note, prevalence scales vary between groups as indicated by the associated legend, emphasizing regional variation among age groups. Cluster weights were re-scaled to sum to 1 within country, then multiplied by the 2010 population of females 20 to 29, ensuring comparability across countries (UN World Population Prospects, 2012). This was done using the following equation for each of the three analysis countries:

$$ {\mathrm{W}}_{\mathrm{new}}=\left({\mathrm{W}}_{\mathrm{DHS}}/{\displaystyle \sum {\mathrm{W}}_{\mathrm{DHS}}}\right)*\mathrm{Pop}{\left(\mathrm{female}\right)}_{20-29} $$

The optimal number of persons surveyed, or N parameter, was calculated using the following formula outlined in Larmarange et al. [22]:

$$ {\mathrm{N}}_{\mathrm{opt}}=14,172\cdotp {n}^{0.419}\cdotp {p}^{0.361}{c}^{0.037}-91.011 $$

where *n* equals number of persons surveyed within the sample, *p* denotes sample prevalence, and *c* specifies number of sample clusters. Because kernel estimator approaches are highly contingent upon bandwidth size, this equation is beneficial in calculating the optimal bandwidth size to produce a surface with sufficiently high resolution smoothness, while also compensating for localized variations. For these analyses, *n* = 9050 total persons surveyed, with *c =* 1252 clusters.

### INLA

A model incorporating both spatial and random effects was used because it produced the lowest deviance information criteria (DIC) as compared to models incorporating a spatial effect or random effect alone. We assumed an uninformative prior distribution, and defined the outcome of interest as following a Bernoulli distribution, with prevalence of adolescent first birth, *p*, ranging between 0 and 1. Covariates of the model included proportion of the sample with rural residences, proportion with no education, and proportion classified as being in the bottom two wealth quintiles.

## Appendix 3: Uncertainty estimates for INLA approximations

**Table 3:** Uncertainty parameters for INLA approximations at less than 16 years old, by country

	Mean	SD	2.5 % quantile	50 % quantile	97.5 % quantile	Mode
TANZANIA						
Aru Meru	0.08451	0.033248	0.032747	0.08007	0.161952	0.071867
Arusha	0.155814	0.056362	0.064061	0.149729	0.282175	0.13709
Babati	0.058121	0.025304	0.019719	0.054604	0.11751	0.048551
Bagamoyo	0.096333	0.032069	0.044821	0.092473	0.170154	0.085443
Bariadi	0.056383	0.020757	0.022529	0.054222	0.102986	0.050172
Biharamulo	0.050484	0.01954	0.019258	0.048248	0.094798	0.043936
Bukoba Rural	0.055073	0.025062	0.017921	0.051191	0.114824	0.044167
Bukombe	0.062469	0.024808	0.023427	0.059385	0.119705	0.05388
Bunda	0.068573	0.029143	0.024077	0.064493	0.137058	0.057262
Chake	0.074684	0.040557	0.021303	0.066404	0.177179	0.053255
Chunya	0.082862	0.027813	0.0384	0.079476	0.147257	0.07369
Dodoma Rural	0.120411	0.037407	0.057911	0.11676	0.20393	0.109849
Dodoma Urban	0.253989	0.079326	0.118003	0.247709	0.425274	0.233964
Geita	0.062487	0.022528	0.025428	0.06029	0.112487	0.055975
Hai	0.077817	0.033216	0.028598	0.072587	0.157267	0.06308
Hanang	0.063946	0.029677	0.020331	0.059329	0.134899	0.051414
Handeni	0.088394	0.032864	0.035916	0.084447	0.163788	0.077126
Igunga	0.09939	0.034739	0.043311	0.09537	0.1788	0.087986
Ilala	0.056176	0.020741	0.022947	0.053745	0.10351	0.049148
Ileje	0.038036	0.022948	0.008444	0.033351	0.09534	0.025397
Ilemela	0.087587	0.0353	0.034481	0.082244	0.171355	0.072191
Iramba	0.065334	0.026536	0.025059	0.061508	0.128181	0.054987
Iringa Rural	0.089756	0.032837	0.04069	0.084593	0.168377	0.075208
Iringa Urban	0.085199	0.044624	0.022765	0.077086	0.194331	0.061876
Kahama	0.110448	0.03185	0.059255	0.106605	0.18314	0.098855
Karagwe	0.042121	0.018663	0.013967	0.039419	0.086061	0.034483
Karatu	0.060075	0.028797	0.0199	0.054853	0.131077	0.046278
Kaskazini ‘A’	0.116896	0.078634	0.019831	0.098334	0.320075	0.064446
Kaskazini ‘B’	0.073364	0.040634	0.020615	0.064997	0.176521	0.052438
Kasulu	0.065958	0.029045	0.022496	0.061572	0.134699	0.053262
Kati	0.07499	0.039878	0.022149	0.066944	0.175614	0.054246
Kibaha	0.084618	0.039698	0.026705	0.078206	0.180446	0.067318
Kibondo	0.046161	0.021507	0.014624	0.042817	0.097395	0.036894
Kigoma Rural	0.078883	0.030973	0.03027	0.074919	0.150693	0.067803
Kigoma Urban	0.225847	0.057116	0.124765	0.222295	0.347181	0.215
Kilindi	0.155555	0.060643	0.068245	0.144661	0.302853	0.123713
Kilolo	0.077967	0.034493	0.028029	0.072185	0.161754	0.06231
Kilombero	0.119978	0.035403	0.06311	0.115653	0.201572	0.107605
Kilosa	0.129224	0.033827	0.073949	0.12542	0.205768	0.117745
Kilwa	0.162477	0.060397	0.068329	0.154281	0.303184	0.13828
Kinondoni	0.087583	0.026503	0.043942	0.084796	0.147213	0.079407
Kisarawe	0.21113	0.082673	0.083622	0.199415	0.404437	0.175685
Kishapu	0.090416	0.028683	0.044903	0.086783	0.156562	0.079776
Kiteto	0.134707	0.049804	0.053746	0.129259	0.247585	0.119235
Kondoa	0.067965	0.02419	0.028576	0.065374	0.122738	0.060834
Kongwa	0.180709	0.066789	0.07221	0.173222	0.331989	0.15839
Korogwe	0.071336	0.03201	0.024234	0.06624	0.148168	0.057226
Kusini	0.092483	0.079195	0.010846	0.069657	0.310113	0.036757
Kwimba	0.083511	0.029469	0.037181	0.079655	0.152053	0.072557
Kyela	0.052566	0.02595	0.014501	0.048516	0.114037	0.040496
Lindi Rural	0.094528	0.038914	0.035467	0.088863	0.186384	0.078425
Lindi Urban	0.082425	0.074563	0.00613	0.060829	0.285073	0.024564
Liwale	0.081193	0.034997	0.030343	0.075399	0.166293	0.065878
Ludewa	0.0646	0.029526	0.021459	0.059871	0.135686	0.051897
Lushoto	0.134968	0.06455	0.043729	0.123168	0.292085	0.099453
Mafia	NA	NA	NA	NA	NA	NA
Magharibi	0.063107	0.031771	0.018615	0.057492	0.140931	0.048167
Magu	0.100567	0.031313	0.05171	0.09628	0.173555	0.087969
Makete	0.065017	0.030634	0.02176	0.05964	0.14007	0.050776
Manyoni	0.061885	0.023697	0.024259	0.059097	0.116333	0.054484
Masasi	0.078111	0.034816	0.028126	0.072093	0.162643	0.061025
Maswa	0.108444	0.041911	0.047504	0.101226	0.2096	0.087304
Mbarali	0.227149	0.056495	0.130459	0.222398	0.350241	0.212217
Mbeya Rural	0.052231	0.023517	0.017635	0.048576	0.108419	0.042347
Mbeya Urban	0.054634	0.030383	0.012569	0.049129	0.128642	0.038603
Mbinga	0.06177	0.027725	0.020141	0.057654	0.12721	0.049833
Mbozi	0.037029	0.019551	0.009416	0.033708	0.084127	0.027464
Mbulu	0.073524	0.038334	0.022257	0.065844	0.170126	0.053574
Meatu	0.060398	0.027398	0.021617	0.055534	0.127436	0.047049
Micheweni	0.106287	0.070963	0.019278	0.089611	0.290222	0.060809
Missungwi	0.064286	0.025384	0.026627	0.060256	0.125336	0.053236
Mjini	0.057105	0.046535	0.006517	0.044838	0.180154	0.024833
Mkoani	0.08658	0.061402	0.014056	0.07158	0.247133	0.046609
Mkuranga	0.09811	0.041137	0.037494	0.091418	0.197063	0.079083
Monduli	0.090703	0.035633	0.033981	0.086528	0.17198	0.078844
Morogoro Rural	0.124807	0.043808	0.056178	0.118938	0.227576	0.108634
Morogoro Urban	0.070508	0.041105	0.014273	0.062863	0.171118	0.048095
Moshi Rural	0.076847	0.02875	0.03239	0.072881	0.144106	0.065472
Moshi Urban	0.105684	0.063682	0.023583	0.091793	0.267687	0.066953
Mpanda	0.088265	0.02989	0.040863	0.084476	0.157583	0.077614
Mpwapwa	0.091513	0.031985	0.040818	0.087463	0.165561	0.079979
Mtwara Rural	0.074234	0.036632	0.021466	0.068093	0.162431	0.056337
Mtwara Urban	0.039085	0.035979	0.002934	0.028852	0.135244	0.011422
Mufindi	0.073355	0.031954	0.026361	0.068213	0.15042	0.059419
Muheza	0.100514	0.048269	0.03344	0.091388	0.219717	0.074827
Muleba	0.045895	0.018884	0.01707	0.043272	0.090232	0.038756
Musoma Rural	0.051797	0.025789	0.014758	0.047495	0.113938	0.03966
Musoma Urban	0.042554	0.038002	0.003284	0.032073	0.143375	0.013593
Mvomero	0.090427	0.040905	0.032314	0.083132	0.190925	0.070622
Mwanga	0.063507	0.034216	0.015928	0.057351	0.147101	0.046369
Nachingwea	0.069241	0.035583	0.020383	0.062545	0.157175	0.050984
Namtumbo	0.076978	0.040271	0.02218	0.069233	0.177404	0.056455
Newala	0.043334	0.025806	0.009525	0.038096	0.107778	0.028872
Ngara	0.038154	0.02518	0.00732	0.032592	0.101987	0.023388
Ngorongoro	0.16146	0.064201	0.062549	0.152414	0.311971	0.134847
Njombe	0.119238	0.033404	0.064717	0.11551	0.194748	0.10797
Nkasi	0.061314	0.033887	0.014469	0.05514	0.144126	0.043701
Nyamagana	0.061473	0.049344	0.006583	0.048598	0.191516	0.026027
Nzega	0.084301	0.029507	0.036095	0.081113	0.151095	0.075262
Rombo	0.064643	0.040042	0.014031	0.055942	0.166466	0.041457
Ruangwa	0.092625	0.046423	0.027134	0.084388	0.206272	0.069917
Rufiji	0.17953	0.064478	0.077116	0.17141	0.328071	0.155462
Rungwe	0.053291	0.023883	0.017966	0.049569	0.110317	0.042963
Same	0.080934	0.0428	0.023753	0.072288	0.188527	0.057678
Sengerema	0.089192	0.025964	0.047098	0.086194	0.148324	0.080394
Serengeti	0.0534	0.025456	0.016814	0.049178	0.115023	0.042114
Shinyanga Rural	0.086924	0.03111	0.037707	0.083009	0.159321	0.076433
Shinyanga Urban	0.107723	0.070857	0.02376	0.089663	0.294683	0.060263
Sikonge	0.125332	0.043218	0.053973	0.120928	0.222227	0.112595
Simanjiro	0.082461	0.026396	0.039015	0.079739	0.142186	0.075199
Singida Rural	0.039779	0.018302	0.012453	0.03709	0.082903	0.03215
Singida Urban	0.037101	0.034594	0.002685	0.02736	0.129159	0.010993
Songea Rural	0.109879	0.041357	0.049097	0.102993	0.210139	0.090941
Songea Urban	0.068551	0.042092	0.01335	0.059892	0.173682	0.04323
Sumbawanga Rural	0.054203	0.026289	0.016109	0.049931	0.117295	0.042193
Sumbawanga Urban	0.076452	0.047216	0.016189	0.066131	0.196413	0.047871
Tabora Urban	0.056579	0.037275	0.011113	0.048194	0.15191	0.034664
Tandahimba	0.044097	0.02691	0.009249	0.038531	0.11147	0.028638
Tanga	0.041107	0.029474	0.005355	0.034308	0.116148	0.020836
Tarime	0.102142	0.036448	0.047715	0.096341	0.188784	0.084803
Temeke	0.065563	0.025048	0.026053	0.06243	0.123188	0.056416
Tunduru	0.07154	0.033423	0.023031	0.065996	0.152274	0.056117
Ukerewe	0.057614	0.025554	0.021499	0.053123	0.120394	0.046
Ulanga	0.147958	0.057004	0.060111	0.139892	0.281558	0.124146
Urambo	0.117271	0.04103	0.051875	0.112219	0.212311	0.103386
Uyui	0.163219	0.037328	0.09924	0.160062	0.245045	0.153703
Wete	0.081566	0.046461	0.022554	0.071585	0.200691	0.056751
KENYA						
Baringo	0.240878	0.054383	0.144752	0.237329	0.357091	0.230062
Bomet	0.074298	0.021292	0.038257	0.072378	0.121435	0.068744
Bungoma	0.044318	0.013862	0.020895	0.043098	0.074839	0.040761
Busia	0.109215	0.037833	0.048304	0.104748	0.195904	0.096441
Embu	0.052192	0.020115	0.021145	0.049416	0.099348	0.04442
Garissa	0.174919	0.058345	0.077717	0.169158	0.30511	0.157844
Homa Bay	0.121284	0.028322	0.072783	0.118894	0.183344	0.114124
Isiolo	0.184606	0.064146	0.0782	0.178157	0.32795	0.165427
Kajiado	0.062478	0.023385	0.026318	0.059254	0.117276	0.05338
Kakamega	0.073914	0.021253	0.037918	0.072007	0.120927	0.0684
Keiyo-Marakwet	0.085423	0.031907	0.036025	0.081026	0.16025	0.073039
Kericho	0.143753	0.046134	0.07013	0.138027	0.249831	0.12684
Kiambu	0.032065	0.011994	0.013079	0.030578	0.059674	0.027807
Kilifi	0.211694	0.034222	0.149241	0.210117	0.283104	0.206938
Kirinyaga	0.054381	0.022489	0.020889	0.050896	0.108097	0.044681
Kisii	0.111926	0.025299	0.068265	0.109908	0.167092	0.10592
Kisumu	0.116235	0.024134	0.073953	0.11452	0.168285	0.111125
Kitui	0.079004	0.024178	0.038306	0.076769	0.132635	0.072523
Kwale	0.20927	0.057402	0.112946	0.203739	0.336636	0.19239
Laikipia	0.143366	0.036594	0.081411	0.140065	0.223988	0.133355
Lamu	0.069575	0.032823	0.024521	0.063373	0.15126	0.053435
Machakos	0.028128	0.012045	0.009587	0.026509	0.056107	0.023427
Makueni	0.060502	0.021534	0.025652	0.058071	0.109455	0.053545
Mandera	0.25923	0.069665	0.134861	0.255266	0.406168	0.247056
Marsabit	0.181116	0.066258	0.072181	0.174216	0.329386	0.160218
Meru	0.088279	0.024018	0.04854	0.085798	0.142115	0.080867
Migori	0.160352	0.041866	0.090546	0.156207	0.253295	0.147457
Mombasa	0.083335	0.028039	0.038232	0.080052	0.147271	0.073709
Murang’a	0.039553	0.015458	0.015478	0.03751	0.075493	0.03378
Nairobi	0.042442	0.009839	0.025189	0.041757	0.063646	0.04044
Nakuru	0.089081	0.016623	0.059299	0.088118	0.124396	0.086253
Nandi	0.097144	0.022557	0.057767	0.095497	0.145967	0.092319
Narok	0.155751	0.042826	0.082072	0.15228	0.249292	0.14548
Nyamira	0.090285	0.028964	0.041713	0.087567	0.154558	0.082338
Nyandarua	0.046398	0.019806	0.016646	0.043449	0.093361	0.038305
Nyeri	0.027712	0.012907	0.008717	0.025693	0.05846	0.021992
Samburu	0.227697	0.079968	0.098865	0.21818	0.410298	0.199088
Siaya	0.10215	0.024268	0.060422	0.100156	0.155278	0.096272
Taita Taveta	0.08037	0.035498	0.030385	0.073912	0.167933	0.062966
Tana River	0.196576	0.063559	0.093364	0.189283	0.341061	0.174739
Tharaka	0.051207	0.02249	0.018089	0.04764	0.105138	0.041395
Trans Nzoia	0.105247	0.028753	0.057245	0.102405	0.169499	0.096891
Turkana	0.194557	0.060246	0.091432	0.189604	0.325898	0.179562
Uasin Gishu	0.079448	0.028699	0.035927	0.075171	0.147412	0.06716
Vihiga	0.078771	0.032048	0.030665	0.073906	0.155337	0.065583
Wajir	0.272639	0.072123	0.146146	0.26767	0.427082	0.257251
West Pokot	0.135679	0.048794	0.056064	0.130346	0.246119	0.12027
UGANDA						
Adjumani	0.11311	0.037516	0.051424	0.109203	0.197941	0.102443
Amolatar	0.225593	0.050985	0.140214	0.220321	0.341249	0.21086
Amuria	0.238159	0.047934	0.15406	0.234663	0.342126	0.227809
Apac	0.213819	0.031924	0.156157	0.212086	0.281308	0.208619
Arua	0.112182	0.027018	0.063934	0.110643	0.169457	0.10775
Bugiri	0.231211	0.038607	0.162263	0.228814	0.313641	0.224012
Bukwa	0.219408	0.068517	0.104826	0.212605	0.37284	0.199432
Bundibugyo	0.13266	0.043817	0.068718	0.125093	0.239329	0.111658
Bushenyi	0.065164	0.016434	0.03603	0.064204	0.100034	0.062452
Busia	0.219604	0.06537	0.109455	0.213315	0.365754	0.20134
Butaleja	0.243775	0.043882	0.166445	0.24064	0.338797	0.23447
Gulu	0.159808	0.02954	0.108047	0.157633	0.223996	0.153486
Hoima	0.202098	0.045815	0.126472	0.197141	0.304882	0.186595
Ibanda	0.0956	0.027404	0.048608	0.093363	0.155774	0.089388
Iganga	0.191756	0.030817	0.13686	0.189777	0.257882	0.18591
Isingiro	0.09311	0.02492	0.050051	0.091152	0.147679	0.087671
Jinja	0.159497	0.032614	0.103422	0.156725	0.231304	0.151341
Kaabong	0.13543	0.048419	0.057197	0.129908	0.245323	0.119069
Kabale	0.059933	0.01985	0.027278	0.057891	0.10452	0.05412
Kabarole	0.111862	0.026323	0.065959	0.109856	0.169522	0.106336
Kaberamaido	0.230658	0.044828	0.153824	0.226718	0.329465	0.218824
Kalangala	0.191447	0.052386	0.1105	0.183805	0.314772	0.169106
Kaliro	0.197438	0.052164	0.107629	0.193003	0.312905	0.184989
Kampala	0.100763	0.016339	0.071005	0.09998	0.135013	0.098459
Kamuli	0.174873	0.030359	0.118624	0.173723	0.23779	0.171576
Kamwenge	0.109061	0.026309	0.064308	0.106684	0.167686	0.102458
Kanungu	0.077251	0.027024	0.033861	0.074074	0.139044	0.068189
Kapchorwa	0.183861	0.050881	0.095939	0.179793	0.295685	0.172536
Kasese	0.095043	0.021976	0.057022	0.093279	0.143265	0.090014
Katakwi	0.206128	0.051711	0.115466	0.20234	0.319273	0.195859
Kayunga	0.173521	0.033484	0.114935	0.170923	0.247056	0.166224
Kibaale	0.135695	0.023504	0.093578	0.134279	0.185924	0.131578
Kiboga	0.148229	0.032892	0.093701	0.144699	0.222909	0.138206
Kiruhura	0.102256	0.031887	0.051447	0.098326	0.175927	0.091278
Kisoro	0.06841	0.026891	0.026763	0.064771	0.131201	0.058109
Kitgum	0.114999	0.037898	0.051325	0.111568	0.199334	0.105807
Koboko	0.089404	0.038708	0.030882	0.08382	0.180833	0.074022
Kotido	0.128391	0.033553	0.070057	0.125901	0.201011	0.121016
Kumi	0.168732	0.030438	0.112402	0.167585	0.231747	0.165449
Kyenjojo	0.117239	0.024688	0.072818	0.115839	0.169915	0.113387
Lira	0.171492	0.032232	0.116239	0.168666	0.242678	0.163165
Luweero	0.134339	0.02803	0.084665	0.132431	0.195078	0.129013
Manafwa	0.165088	0.042104	0.090906	0.162242	0.255736	0.156845
Masaka	0.135582	0.028793	0.086288	0.133073	0.199104	0.128183
Masindi	0.16548	0.027297	0.117305	0.163553	0.224586	0.159836
Mayuge	0.23409	0.050814	0.146076	0.229909	0.345818	0.221896
Mbale	0.164225	0.035585	0.102562	0.161349	0.242471	0.156037
Mbarara	0.057829	0.01808	0.027105	0.056364	0.097351	0.053811
Mityana	0.131343	0.029062	0.079916	0.129349	0.19444	0.12588
Moroto	0.162426	0.053144	0.075508	0.156686	0.281999	0.145115
Moyo	0.086994	0.042507	0.025361	0.080113	0.189049	0.067849
Mpigi	0.158659	0.032219	0.102937	0.156043	0.229332	0.151032
Mubende	0.120171	0.025597	0.073614	0.118922	0.174137	0.116754
Mukono	0.151077	0.024578	0.107426	0.149451	0.203995	0.146319
Nakapiripirit	0.16346	0.039934	0.094645	0.160235	0.250693	0.153917
Nakaseke	0.161878	0.040869	0.092914	0.157973	0.253598	0.151006
Nakasongola	0.13504	0.031366	0.080687	0.132483	0.204359	0.128063
Nebbi	0.134151	0.031345	0.079337	0.131836	0.202307	0.127506
Ntungamo	0.068901	0.020033	0.034236	0.067474	0.11206	0.064857
Pader	0.165599	0.038104	0.096614	0.163632	0.246259	0.160209
Pallisa	0.228123	0.03459	0.166691	0.225832	0.302438	0.221251
Rakai	0.155973	0.03306	0.100209	0.152801	0.229606	0.146554
Rukungiri	0.053825	0.019062	0.022763	0.05183	0.096638	0.048135
Sembabule	0.12082	0.030583	0.069114	0.117959	0.18917	0.112849
Sironko	0.14112	0.035489	0.078046	0.138931	0.217349	0.135243
Soroti	0.197322	0.039985	0.12383	0.195579	0.281111	0.192532
Tororo	0.20023	0.039523	0.129513	0.19783	0.284652	0.193218
Wakiso	0.100557	0.016272	0.070805	0.099806	0.134632	0.098363
Yumbe	0.122695	0.037781	0.058724	0.119337	0.206169	0.11303

**Table 4:** Uncertainty parameters for INLA approximations at 16 to 17 years old, by country

	Mean	SD	2.5 % quantile	50 % quantile	97.5 % quantile	Mode
TANZANIA						
Aru Meru	0.196509	0.037348	0.125533	0.19601	0.272189	0.196361
Arusha	0.133765	0.042022	0.060398	0.131161	0.223349	0.126908
Babati	0.207838	0.034687	0.140043	0.208022	0.277337	0.21001
Bagamoyo	0.26831	0.03743	0.201949	0.265362	0.350468	0.259738
Bariadi	0.280316	0.035745	0.21612	0.27794	0.357424	0.273262
Biharamulo	0.274124	0.034984	0.209486	0.272433	0.348281	0.269328
Bukoba Rural	0.257286	0.041986	0.178739	0.255629	0.345618	0.252866
Bukombe	0.288836	0.040081	0.217337	0.285867	0.376504	0.280343
Bunda	0.285901	0.044351	0.209322	0.28172	0.38443	0.273384
Chake	0.243117	0.048247	0.157906	0.239178	0.351236	0.233095
Chunya	0.23003	0.031322	0.168578	0.230032	0.293035	0.230948
Dodoma Rural	0.269781	0.038891	0.19592	0.268835	0.34958	0.267424
Dodoma Urban	0.195712	0.053961	0.097036	0.193869	0.307643	0.192273
Geita	0.295699	0.035165	0.229186	0.294592	0.368518	0.292567
Hai	0.199233	0.039333	0.12587	0.198075	0.280261	0.196593
Hanang	0.202052	0.038051	0.127773	0.202592	0.27672	0.206117
Handeni	0.250436	0.039705	0.179614	0.247617	0.337187	0.242567
Igunga	0.300037	0.042392	0.22374	0.297364	0.391368	0.292332
Ilala	0.20593	0.032415	0.144661	0.205089	0.272316	0.20372
Ileje	0.188653	0.041386	0.111773	0.187384	0.275074	0.186494
Ilemela	0.231754	0.042486	0.157721	0.228323	0.324979	0.221678
Iramba	0.222817	0.035408	0.15464	0.222398	0.294765	0.222506
Iringa Rural	0.221798	0.034556	0.158664	0.219992	0.295582	0.216921
Iringa Urban	0.157236	0.043423	0.077981	0.155894	0.247113	0.15554
Kahama	0.276106	0.034467	0.214671	0.273618	0.350837	0.268631
Karagwe	0.23927	0.036606	0.169041	0.238612	0.314021	0.23789
Karatu	0.208322	0.038773	0.136067	0.206894	0.290023	0.205138
Kaskazini ‘A’	0.244126	0.069652	0.119378	0.240021	0.396207	0.234965
Kaskazini ‘B’	0.236907	0.045793	0.153483	0.234183	0.337319	0.230496
Kasulu	0.179143	0.039956	0.103977	0.178661	0.25903	0.179427
Kati	0.241886	0.046725	0.158248	0.238605	0.344937	0.233502
Kibaha	0.266113	0.047097	0.184018	0.261764	0.371875	0.254185
Kibondo	0.215485	0.037535	0.143165	0.215245	0.290956	0.215982
Kigoma Rural	0.235916	0.038157	0.162074	0.235618	0.313198	0.23614
Kigoma Urban	0.233238	0.048968	0.145814	0.230205	0.337843	0.224192
Kilindi	0.257961	0.04029	0.182387	0.256342	0.343599	0.254078
Kilolo	0.240477	0.042785	0.165591	0.237008	0.33512	0.230866
Kilombero	0.269854	0.034629	0.207868	0.267456	0.344997	0.26288
Kilosa	0.265969	0.032926	0.209172	0.262891	0.33872	0.256459
Kilwa	0.332065	0.053337	0.232679	0.329867	0.443959	0.325801
Kinondoni	0.241881	0.035433	0.179091	0.239426	0.318095	0.234315
Kisarawe	0.354954	0.061949	0.239323	0.352682	0.483564	0.348349
Kishapu	0.225676	0.032372	0.166581	0.223908	0.294896	0.220723
Kiteto	0.308573	0.046995	0.219653	0.30719	0.405962	0.305024
Kondoa	0.229844	0.032321	0.166031	0.23014	0.293766	0.231818
Kongwa	0.386599	0.065492	0.273332	0.380932	0.52921	0.36811
Korogwe	0.211879	0.039837	0.137028	0.210726	0.295	0.209685
Kusini	0.244388	0.076257	0.118422	0.235487	0.422577	0.22195
Kwimba	0.247029	0.036497	0.180037	0.24514	0.324938	0.241831
Kyela	0.259327	0.045268	0.176849	0.256989	0.355256	0.252633
Lindi Rural	0.300043	0.047873	0.211565	0.297892	0.400896	0.293942
Lindi Urban	0.237292	0.081907	0.097112	0.230343	0.420801	0.219958
Liwale	0.288745	0.047066	0.210112	0.283453	0.394958	0.27266
Ludewa	0.222916	0.038856	0.148692	0.22215	0.3031	0.221723
Lushoto	0.191085	0.047511	0.102741	0.190127	0.288144	0.190734
Mafia	NA	NA	NA	NA	NA	NA
Magharibi	0.223738	0.042254	0.149528	0.220176	0.318467	0.214522
Magu	0.236964	0.031192	0.177243	0.236218	0.301384	0.235156
Makete	0.182905	0.035085	0.11731	0.181854	0.256196	0.181015
Manyoni	0.224272	0.031517	0.163664	0.223624	0.290071	0.223313
Masasi	0.249642	0.04572	0.168875	0.246394	0.348746	0.240091
Maswa	0.201219	0.034891	0.132557	0.201667	0.269704	0.204099
Mbarali	0.27185	0.043815	0.186819	0.271914	0.357674	0.273
Mbeya Rural	0.202081	0.035117	0.13483	0.201675	0.273414	0.202085
Mbeya Urban	0.193593	0.043258	0.115444	0.191235	0.286375	0.18766
Mbinga	0.25205	0.04487	0.171092	0.249409	0.347874	0.244286
Mbozi	0.201996	0.037507	0.134538	0.199592	0.283949	0.195866
Mbulu	0.194358	0.041119	0.115145	0.194579	0.276277	0.197773
Meatu	0.198127	0.038824	0.126864	0.196496	0.279111	0.193623
Micheweni	0.261553	0.06763	0.140521	0.257069	0.411278	0.251247
Missungwi	0.211674	0.034433	0.145554	0.211159	0.281565	0.210773
Mjini	0.22895	0.068146	0.118833	0.220075	0.388887	0.205207
Mkoani	0.253887	0.067586	0.137631	0.247408	0.408209	0.237699
Mkuranga	0.238666	0.043086	0.155985	0.238	0.326764	0.237776
Monduli	0.226989	0.039706	0.149489	0.227488	0.304064	0.230584
Morogoro Rural	0.310657	0.042959	0.233688	0.307611	0.404152	0.301802
Morogoro Urban	0.308678	0.067421	0.198209	0.30056	0.460218	0.281852
Moshi Rural	0.161898	0.032192	0.100926	0.161585	0.226233	0.162294
Moshi Urban	0.176943	0.050366	0.089399	0.173235	0.288652	0.168377
Mpanda	0.238624	0.034022	0.172744	0.238252	0.308008	0.23838
Mpwapwa	0.251599	0.037727	0.18373	0.249057	0.333741	0.244522
Mtwara Rural	0.25288	0.052499	0.154304	0.251502	0.360272	0.249504
Mtwara Urban	0.176684	0.060687	0.07318	0.171737	0.310283	0.163507
Mufindi	0.220957	0.038654	0.150476	0.218817	0.304362	0.215472
Muheza	0.218341	0.045249	0.135946	0.215977	0.315635	0.212621
Muleba	0.264975	0.038474	0.198306	0.261558	0.350241	0.255005
Musoma Rural	0.310468	0.054925	0.217471	0.305154	0.431082	0.292644
Musoma Urban	0.280474	0.075826	0.152574	0.272539	0.451048	0.25687
Mvomero	0.265121	0.047086	0.1836	0.260804	0.370123	0.25279
Mwanga	0.19923	0.043155	0.121276	0.196747	0.293177	0.193463
Nachingwea	0.295656	0.058257	0.197968	0.289581	0.42591	0.276892
Namtumbo	0.295401	0.058444	0.20061	0.287838	0.428844	0.271808
Newala	0.257576	0.053883	0.166236	0.252223	0.378209	0.241749
Ngara	0.222475	0.048966	0.132231	0.2204	0.326721	0.218
Ngorongoro	0.281995	0.055274	0.175516	0.281749	0.391677	0.282641
Njombe	0.203965	0.030988	0.143162	0.20426	0.26448	0.205881
Nkasi	0.232013	0.048542	0.141767	0.2302	0.334771	0.228222
Nyamagana	0.209599	0.065731	0.097889	0.203408	0.357919	0.193147
Nzega	0.296275	0.040798	0.224255	0.293089	0.385367	0.286743
Rombo	0.184824	0.047905	0.097631	0.182973	0.285125	0.181202
Ruangwa	0.30852	0.056267	0.207834	0.304531	0.431385	0.297245
Rufiji	0.311661	0.052857	0.210944	0.310516	0.419432	0.308709
Rungwe	0.20817	0.036833	0.138726	0.207191	0.284286	0.206083
Same	0.215007	0.046861	0.132468	0.211508	0.318576	0.205941
Sengerema	0.273206	0.033236	0.213188	0.271143	0.344401	0.266978
Serengeti	0.277872	0.047463	0.198734	0.272467	0.385394	0.261753
Shinyanga Rural	0.279379	0.037031	0.211514	0.277258	0.359297	0.27362
Shinyanga Urban	0.27475	0.072233	0.163286	0.263313	0.445253	0.241125
Sikonge	0.327621	0.04698	0.241454	0.325304	0.427039	0.320965
Simanjiro	0.212701	0.029266	0.154675	0.213265	0.269442	0.215746
Singida Rural	0.198857	0.033611	0.135167	0.198073	0.268006	0.197276
Singida Urban	0.240093	0.071055	0.125583	0.230718	0.40549	0.213582
Songea Rural	0.251444	0.037836	0.184815	0.248301	0.335304	0.24264
Songea Urban	0.265447	0.06621	0.1552	0.258286	0.413625	0.24286
Sumbawanga Rural	0.227018	0.041357	0.149743	0.225598	0.3138	0.223875
Sumbawanga Urban	0.184311	0.050529	0.092549	0.182351	0.290738	0.18099
Tabora Urban	0.201293	0.049602	0.115828	0.196834	0.313517	0.190086
Tandahimba	0.240206	0.05097	0.148492	0.237078	0.350376	0.231754
Tanga	0.174723	0.049256	0.087782	0.171509	0.280986	0.166053
Tarime	0.275796	0.038902	0.208854	0.27217	0.361716	0.264563
Temeke	0.24118	0.039602	0.169652	0.238943	0.325245	0.234483
Tunduru	0.32394	0.060874	0.224239	0.316972	0.458465	0.297535
Ukerewe	0.254646	0.041224	0.183826	0.250775	0.34669	0.243471
Ulanga	0.284495	0.05023	0.195087	0.280764	0.394548	0.27392
Urambo	0.299555	0.041115	0.221655	0.298291	0.385179	0.296344
Uyui	0.309569	0.035275	0.242862	0.3086	0.381878	0.306778
Wete	0.240304	0.047343	0.152984	0.237901	0.343737	0.235273
KENYA						
Baringo	0.280847	0.04762	0.194049	0.278468	0.381173	0.273821
Bomet	0.154695	0.029074	0.099997	0.15411	0.213329	0.153406
Bungoma	0.226183	0.029655	0.172687	0.224565	0.288623	0.221112
Busia	0.251005	0.047861	0.168039	0.24696	0.356639	0.239223
Embu	0.16401	0.032839	0.105113	0.162015	0.234561	0.158468
Garissa	0.186178	0.046071	0.107778	0.181986	0.288814	0.174287
Homa Bay	0.257432	0.038098	0.190352	0.254843	0.338476	0.248788
Isiolo	0.20238	0.050699	0.111965	0.199328	0.310853	0.193886
Kajiado	0.169426	0.037165	0.108594	0.165161	0.2537	0.156486
Kakamega	0.184479	0.030161	0.127661	0.183689	0.246092	0.18238
Keiyo-Marakwet	0.187146	0.040869	0.110663	0.186114	0.270778	0.185035
Kericho	0.222246	0.04376	0.144106	0.219377	0.316989	0.214217
Kiambu	0.139956	0.024961	0.095689	0.138308	0.193589	0.135105
Kilifi	0.165524	0.026199	0.117482	0.16434	0.220356	0.162073
Kirinyaga	0.170843	0.036569	0.105642	0.16852	0.249576	0.164356
Kisii	0.164906	0.0263	0.115552	0.164135	0.218876	0.162807
Kisumu	0.197797	0.027285	0.148154	0.196414	0.255254	0.193661
Kitui	0.20501	0.034809	0.139341	0.204165	0.275859	0.202785
Kwale	0.202566	0.041888	0.12835	0.199653	0.293649	0.194419
Laikipia	0.143303	0.029133	0.092338	0.141125	0.206731	0.136993
Lamu	0.148656	0.037662	0.084011	0.145374	0.233092	0.140249
Machakos	0.133579	0.025963	0.086879	0.132086	0.188925	0.129323
Makueni	0.189024	0.035255	0.122924	0.188048	0.261203	0.186529
Mandera	0.216108	0.052223	0.124273	0.212502	0.328541	0.205404
Marsabit	0.185643	0.051589	0.096705	0.18151	0.298419	0.173656
Meru	0.133552	0.024667	0.088093	0.132544	0.18496	0.130764
Migori	0.25658	0.042892	0.182426	0.253011	0.350088	0.245338
Mombasa	0.120001	0.029099	0.070998	0.117209	0.184919	0.111838
Murang’a	0.138139	0.027697	0.087983	0.136667	0.196917	0.134041
Nairobi	0.079662	0.013225	0.055489	0.079067	0.107241	0.077901
Nakuru	0.226384	0.023992	0.181688	0.225554	0.275766	0.22388
Nandi	0.200027	0.02865	0.14611	0.199223	0.258634	0.19774
Narok	0.268461	0.045691	0.184903	0.266249	0.364675	0.262019
Nyamira	0.239471	0.040536	0.163534	0.238237	0.322634	0.235976
Nyandarua	0.144239	0.032221	0.087981	0.141718	0.215341	0.137437
Nyeri	0.109217	0.026222	0.062302	0.107723	0.165022	0.105092
Samburu	0.169876	0.047541	0.091843	0.164586	0.27862	0.155232
Siaya	0.241909	0.033918	0.181333	0.239823	0.313943	0.235346
Taita Taveta	0.140577	0.036839	0.081648	0.135836	0.226791	0.127791
Tana River	0.230908	0.049846	0.141086	0.227987	0.337919	0.222947
Tharaka	0.165256	0.037629	0.09728	0.163247	0.24534	0.159941
Trans Nzoia	0.18405	0.031788	0.126529	0.182279	0.251775	0.178977
Turkana	0.209598	0.050717	0.120595	0.206017	0.319146	0.199059
Uasin Gishu	0.174314	0.036557	0.113315	0.170483	0.256701	0.163044
Vihiga	0.18573	0.040564	0.114522	0.182588	0.275562	0.177464
Wajir	0.172051	0.045473	0.093623	0.168385	0.271562	0.161383
West Pokot	0.295851	0.058226	0.195713	0.290656	0.424829	0.280531
UGANDA						
Adjumani	0.175812	0.041541	0.099922	0.174042	0.263253	0.171772
Amolatar	0.295131	0.046315	0.211452	0.292174	0.395651	0.286955
Amuria	0.288093	0.044457	0.206507	0.285951	0.381948	0.281909
Apac	0.306544	0.034361	0.244056	0.304774	0.378765	0.301019
Arua	0.19952	0.033153	0.136504	0.198907	0.266451	0.198044
Bugiri	0.25021	0.034443	0.185703	0.249011	0.321731	0.246882
Bukwa	0.294421	0.066648	0.176195	0.289679	0.439674	0.280915
Bundibugyo	0.16406	0.036443	0.101364	0.160862	0.245772	0.15568
Bushenyi	0.16965	0.025727	0.120518	0.169254	0.221428	0.1688
Busia	0.262334	0.059642	0.152111	0.25981	0.388269	0.256005
Butaleja	0.266473	0.038351	0.19279	0.265772	0.344559	0.264765
Gulu	0.204569	0.028873	0.151185	0.203352	0.26502	0.201141
Hoima	0.275879	0.042515	0.200089	0.272968	0.367963	0.26741
Ibanda	0.193311	0.036343	0.125572	0.192067	0.268872	0.190251
Iganga	0.241874	0.030088	0.186977	0.240286	0.305747	0.237264
Isingiro	0.21005	0.03524	0.14482	0.20858	0.284098	0.206125
Jinja	0.162851	0.02783	0.110566	0.162001	0.220356	0.160706
Kaabong	0.279691	0.062994	0.164758	0.276797	0.411265	0.27113
Kabale	0.131677	0.02886	0.079616	0.130185	0.192783	0.127738
Kabarole	0.235756	0.037167	0.172085	0.232288	0.318662	0.225709
Kaberamaido	0.255666	0.038436	0.186158	0.253376	0.338193	0.249129
Kalangala	0.240683	0.044053	0.155996	0.240023	0.330082	0.23946
Kaliro	0.322879	0.05758	0.222808	0.317877	0.450392	0.308308
Kampala	0.1463	0.018959	0.11143	0.1455	0.185736	0.143918
Kamuli	0.3072	0.035382	0.241909	0.305667	0.381029	0.302551
Kamwenge	0.173837	0.029778	0.118489	0.172718	0.236122	0.171021
Kanungu	0.142153	0.035235	0.078944	0.140306	0.216483	0.137125
Kapchorwa	0.319496	0.05916	0.217524	0.314155	0.450912	0.303845
Kasese	0.184621	0.028732	0.132596	0.183003	0.245948	0.180036
Katakwi	0.281408	0.04986	0.190007	0.278786	0.388244	0.27439
Kayunga	0.269708	0.039045	0.204397	0.265476	0.35745	0.25668
Kibaale	0.202331	0.026128	0.151897	0.202004	0.254917	0.201603
Kiboga	0.202935	0.03393	0.136021	0.2032	0.269622	0.204569
Kiruhura	0.211759	0.043965	0.131228	0.209828	0.303797	0.206495
Kisoro	0.123258	0.037053	0.058134	0.121115	0.201379	0.117167
Kitgum	0.201121	0.045228	0.124454	0.196633	0.303879	0.189183
Koboko	0.144704	0.044489	0.067925	0.141412	0.242108	0.136499
Kotido	0.289505	0.0463	0.204752	0.287421	0.386064	0.283234
Kumi	0.211406	0.030656	0.152514	0.211001	0.272906	0.210465
Kyenjojo	0.231953	0.031623	0.171953	0.231083	0.297183	0.22967
Lira	0.224915	0.031784	0.168933	0.22252	0.294243	0.217902
Luweero	0.208878	0.03155	0.153184	0.206488	0.278046	0.202092
Manafwa	0.309145	0.050949	0.217306	0.306168	0.417667	0.300246
Masaka	0.17718	0.028633	0.124735	0.175766	0.237857	0.173266
Masindi	0.241684	0.028528	0.188356	0.240582	0.301269	0.238533
Mayuge	0.262007	0.044323	0.177198	0.261046	0.353025	0.259783
Mbale	0.186578	0.032558	0.124482	0.18597	0.252858	0.185356
Mbarara	0.13194	0.026732	0.082436	0.131008	0.187587	0.129857
Mityana	0.231824	0.035	0.169826	0.229204	0.308549	0.224418
Moroto	0.290668	0.063144	0.176012	0.287654	0.422413	0.28145
Moyo	0.167603	0.055279	0.072914	0.163252	0.289476	0.15663
Mpigi	0.28604	0.039039	0.21629	0.283549	0.369626	0.278494
Mubende	0.303667	0.041597	0.232332	0.299981	0.394476	0.291517
Mukono	0.202176	0.025175	0.153827	0.201733	0.253242	0.201042
Nakapiripirit	0.256112	0.044342	0.17519	0.254006	0.349129	0.249915
Nakaseke	0.26777	0.045475	0.181674	0.266392	0.36197	0.264083
Nakasongola	0.217111	0.036363	0.15506	0.213594	0.298868	0.207214
Nebbi	0.231648	0.038219	0.164597	0.228737	0.315013	0.223072
Ntungamo	0.182579	0.030895	0.125081	0.181482	0.246825	0.179781
Pader	0.308002	0.046204	0.226472	0.304571	0.408495	0.297776
Pallisa	0.277776	0.033357	0.218338	0.27554	0.349563	0.271031
Rakai	0.222688	0.033674	0.158241	0.222082	0.290931	0.221214
Rukungiri	0.114588	0.027047	0.06602	0.113188	0.171923	0.111077
Sembabule	0.27527	0.044699	0.197381	0.271669	0.373551	0.264871
Sironko	0.226162	0.039114	0.155244	0.223879	0.310571	0.220066
Soroti	0.288031	0.041096	0.212374	0.286052	0.375039	0.28243
Tororo	0.202915	0.034988	0.136224	0.202294	0.273764	0.201609
Wakiso	0.143636	0.018127	0.109687	0.14306	0.180902	0.141975
Yumbe	0.203297	0.045624	0.118157	0.2021	0.296335	0.200578

**Table 5:** Uncertainty parameters for INLA approximations at 18 to 19 years old, by country

	Mean	SD	2.5 % quantile	50 % quantile	97.5 % quantile	Mode
TANZANIA						
Aru Meru	0.31247	0.04528	0.222446	0.312955	0.402359	0.315313
Arusha	0.320957	0.047781	0.231205	0.319149	0.421037	0.315945
Babati	0.320572	0.048529	0.223845	0.320907	0.418848	0.323048
Bagamoyo	0.322634	0.044197	0.239555	0.320698	0.416905	0.317673
Bariadi	0.35789	0.041725	0.275859	0.357644	0.441946	0.357564
Biharamulo	0.389236	0.050479	0.303231	0.384839	0.496441	0.369746
Bukoba Rural	0.308505	0.045326	0.217004	0.310255	0.393956	0.31657
Bukombe	0.33162	0.045099	0.242138	0.331723	0.422221	0.332768
Bunda	0.351332	0.050213	0.262914	0.346745	0.462399	0.337548
Chake	0.324251	0.053485	0.218542	0.324267	0.433279	0.3259
Chunya	0.342701	0.048369	0.255487	0.339026	0.448738	0.33213
Dodoma Rural	0.300551	0.04737	0.206123	0.301682	0.39133	0.305947
Dodoma Urban	0.303218	0.047093	0.213784	0.301634	0.402504	0.29945
Geita	0.357681	0.047054	0.276189	0.35363	0.459232	0.343455
Hai	0.305844	0.050641	0.207209	0.305622	0.407671	0.306414
Hanang	0.349456	0.051234	0.249382	0.348572	0.456064	0.347888
Handeni	0.280844	0.042624	0.199411	0.279724	0.36971	0.278393
Igunga	0.323477	0.049457	0.224879	0.324255	0.41992	0.327118
Ilala	0.293633	0.038309	0.219933	0.293033	0.371193	0.292218
Ileje	0.407668	0.059398	0.293701	0.405634	0.532622	0.402045
Ilemela	0.319438	0.04771	0.229694	0.3176	0.419568	0.31432
Iramba	0.392154	0.053863	0.287441	0.391054	0.503802	0.389579
Iringa Rural	0.324951	0.050746	0.223151	0.326226	0.422677	0.330652
Iringa Urban	0.322841	0.050985	0.226457	0.321032	0.430003	0.318166
Kahama	0.334012	0.039984	0.25555	0.33375	0.414748	0.333716
Karagwe	0.307798	0.043936	0.219384	0.30955	0.389734	0.316306
Karatu	0.323795	0.055457	0.211828	0.325785	0.429553	0.332663
Kaskazini ‘A’	0.314255	0.055755	0.209951	0.311748	0.434068	0.308302
Kaskazini ‘B’	0.341842	0.05688	0.230927	0.340914	0.460589	0.340613
Kasulu	0.33411	0.043105	0.248217	0.33455	0.419379	0.336419
Kati	0.330826	0.055642	0.221973	0.330352	0.445166	0.330902
Kibaha	0.32074	0.051772	0.22628	0.317154	0.435055	0.311698
Kibondo	0.411865	0.054861	0.309771	0.408951	0.529052	0.403384
Kigoma Rural	0.317088	0.046603	0.223192	0.318162	0.408419	0.321884
Kigoma Urban	0.307188	0.044843	0.226339	0.304423	0.402968	0.298678
Kilindi	0.35754	0.053155	0.256236	0.355552	0.471022	0.352956
Kilolo	0.332595	0.055284	0.224074	0.332463	0.444504	0.333633
Kilombero	0.309342	0.044935	0.22539	0.307265	0.40531	0.303885
Kilosa	0.330452	0.040008	0.252616	0.329843	0.412494	0.329201
Kilwa	0.309914	0.051907	0.213165	0.307594	0.420529	0.30395
Kinondoni	0.25156	0.038762	0.176207	0.251968	0.326002	0.254319
Kisarawe	0.232913	0.051773	0.13918	0.230108	0.344195	0.225937
Kishapu	0.3899	0.043871	0.306936	0.388367	0.481093	0.38529
Kiteto	0.301865	0.053485	0.206668	0.297842	0.419698	0.290874
Kondoa	0.40771	0.05339	0.317114	0.402091	0.524251	0.387472
Kongwa	0.249474	0.04948	0.156935	0.247845	0.352756	0.245766
Korogwe	0.344007	0.051197	0.241364	0.344784	0.44592	0.348011
Kusini	0.324518	0.059917	0.209287	0.323308	0.449355	0.322694
Kwimba	0.404527	0.047901	0.314196	0.402691	0.504569	0.399006
Kyela	0.382878	0.062751	0.274887	0.37807	0.515081	0.362459
Lindi Rural	0.317582	0.051423	0.216102	0.317626	0.421531	0.319062
Lindi Urban	0.297252	0.060573	0.193838	0.290928	0.435193	0.279932
Liwale	0.361727	0.055502	0.252605	0.36106	0.477321	0.361237
Ludewa	0.398258	0.058641	0.29686	0.392386	0.52793	0.379703
Lushoto	0.370783	0.054612	0.262617	0.370728	0.481792	0.371916
Mafia	0.418937	0.202223	0.077369	0.408923	0.811563	0.363045
Magharibi	0.304103	0.050092	0.206666	0.303349	0.408052	0.303157
Magu	0.398935	0.046363	0.31444	0.396037	0.497959	0.38978
Makete	0.361404	0.056417	0.258844	0.357308	0.484915	0.349655
Manyoni	0.381837	0.052815	0.289005	0.376788	0.499074	0.366909
Masasi	0.422123	0.055394	0.316616	0.420517	0.536465	0.41739
Maswa	0.419944	0.053544	0.324061	0.415834	0.535871	0.406956
Mbarali	0.240254	0.042716	0.15777	0.240088	0.32527	0.240885
Mbeya Rural	0.349545	0.054988	0.23671	0.351887	0.454645	0.35868
Mbeya Urban	0.341603	0.054676	0.247483	0.336441	0.462032	0.324975
Mbinga	0.377534	0.047076	0.291958	0.37483	0.477224	0.368886
Mbozi	0.382723	0.051179	0.290924	0.378882	0.493616	0.370659
Mbulu	0.360172	0.054606	0.249749	0.361279	0.46844	0.365092
Meatu	0.484529	0.063137	0.365534	0.48243	0.614552	0.477836
Micheweni	0.310312	0.053132	0.208951	0.308525	0.423968	0.306779
Missungwi	0.438877	0.052789	0.33971	0.436733	0.54925	0.432304
Mjini	0.311552	0.055338	0.205706	0.310105	0.427551	0.308673
Mkoani	0.304824	0.052504	0.204806	0.303005	0.41722	0.301175
Mkuranga	0.341552	0.04888	0.245767	0.341132	0.441977	0.341617
Monduli	0.302651	0.048843	0.212642	0.299887	0.408599	0.295499
Morogoro Rural	0.288269	0.04942	0.190288	0.288905	0.387027	0.292335
Morogoro Urban	0.283912	0.048085	0.194337	0.281699	0.386806	0.278391
Moshi Rural	0.331276	0.043704	0.247612	0.330142	0.421906	0.328424
Moshi Urban	0.280013	0.052452	0.177515	0.280093	0.385913	0.282389
Mpanda	0.332515	0.043743	0.246806	0.331999	0.42211	0.331616
Mpwapwa	0.354461	0.045545	0.264081	0.354579	0.445863	0.355706
Mtwara Rural	0.411653	0.056407	0.312657	0.407024	0.533245	0.395636
Mtwara Urban	0.31607	0.055961	0.210297	0.314234	0.434018	0.312098
Mufindi	0.348416	0.053256	0.242122	0.348992	0.454159	0.351452
Muheza	0.353018	0.052753	0.248011	0.353284	0.459715	0.35534
Muleba	0.353914	0.052347	0.246613	0.356374	0.451977	0.363925
Musoma Rural	0.328478	0.050416	0.232015	0.327162	0.433212	0.325278
Musoma Urban	0.357358	0.057899	0.257269	0.351412	0.486926	0.339374
Mvomero	0.350595	0.053806	0.247828	0.348887	0.464302	0.346777
Mwanga	0.338238	0.053669	0.244724	0.333082	0.457888	0.323244
Nachingwea	0.384357	0.062082	0.258301	0.386259	0.503489	0.391957
Namtumbo	0.343734	0.05499	0.233163	0.344718	0.453117	0.348494
Newala	0.371835	0.053678	0.264568	0.372291	0.478828	0.374268
Ngara	0.405847	0.059226	0.289804	0.404973	0.52779	0.403982
Ngorongoro	0.341063	0.060646	0.233888	0.336436	0.473539	0.327454
Njombe	0.328978	0.040338	0.251803	0.327942	0.412159	0.326146
Nkasi	0.33276	0.050179	0.240071	0.329651	0.441885	0.32423
Nyamagana	0.324445	0.056363	0.21872	0.322055	0.444463	0.318457
Nzega	0.328593	0.043638	0.243453	0.328106	0.417427	0.327736
Rombo	0.31995	0.058083	0.208802	0.318711	0.440228	0.317681
Ruangwa	0.376173	0.058587	0.271501	0.371296	0.505666	0.3623
Rufiji	0.2921	0.054206	0.191519	0.28977	0.406858	0.286109
Rungwe	0.382463	0.049938	0.289838	0.37991	0.488377	0.374745
Same	0.318502	0.056007	0.207328	0.319368	0.428657	0.323305
Sengerema	0.374041	0.040065	0.29838	0.372598	0.457435	0.369719
Serengeti	0.359979	0.055163	0.2467	0.362412	0.464623	0.369536
Shinyanga Rural	0.379144	0.054588	0.279058	0.37551	0.498122	0.36901
Shinyanga Urban	0.389361	0.063204	0.259997	0.391722	0.510189	0.398594
Sikonge	0.256687	0.046268	0.168601	0.255693	0.351674	0.254616
Simanjiro	0.314515	0.04894	0.217709	0.314443	0.415113	0.31583
Singida Rural	0.416127	0.050664	0.320068	0.414325	0.521921	0.410876
Singida Urban	0.365034	0.055703	0.257339	0.363681	0.481485	0.362041
Songea Rural	0.306958	0.048894	0.208466	0.308014	0.403922	0.311768
Songea Urban	0.348621	0.051272	0.251851	0.346542	0.457024	0.342991
Sumbawanga Rural	0.404125	0.054051	0.305213	0.400599	0.520657	0.393485
Sumbawanga Urban	0.369092	0.055385	0.267382	0.365453	0.489453	0.358691
Tabora Urban	0.320985	0.056572	0.213545	0.319016	0.440928	0.316456
Tandahimba	0.442488	0.062256	0.336057	0.436146	0.578152	0.419318
Tanga	0.333769	0.049775	0.243391	0.330563	0.440998	0.324208
Tarime	0.363744	0.045881	0.27613	0.3622	0.460002	0.359564
Temeke	0.285562	0.037889	0.212228	0.285161	0.361871	0.284871
Tunduru	0.367182	0.051299	0.264543	0.367676	0.469548	0.36981
Ukerewe	0.366894	0.058014	0.250484	0.367452	0.483364	0.369833
Ulanga	0.292102	0.048153	0.197546	0.292069	0.389465	0.293397
Urambo	0.310597	0.051835	0.217527	0.306811	0.424595	0.300245
Uyui	0.259528	0.038336	0.184848	0.259474	0.335507	0.260072
Wete	0.361817	0.056489	0.251176	0.360771	0.480695	0.360186
KENYA						
Baringo	0.279899	0.043984	0.19746	0.278453	0.370775	0.275833
Bomet	0.276945	0.034905	0.213838	0.274963	0.35096	0.270856
Bungoma	0.224521	0.026764	0.175078	0.223388	0.280368	0.221149
Busia	0.232091	0.040469	0.156074	0.230725	0.316654	0.228721
Embu	0.214275	0.037031	0.142574	0.21418	0.287734	0.214924
Garissa	0.201187	0.043794	0.123553	0.19825	0.295992	0.193012
Homa Bay	0.256105	0.032846	0.196978	0.254133	0.326086	0.250078
Isiolo	0.274116	0.05515	0.175758	0.270557	0.393064	0.263994
Kajiado	0.224215	0.038579	0.157759	0.220821	0.309477	0.214157
Kakamega	0.284895	0.035038	0.221014	0.283082	0.358806	0.279374
Keiyo-Marakwet	0.263977	0.04455	0.177366	0.263777	0.353027	0.264284
Kericho	0.214343	0.039116	0.140096	0.213422	0.294804	0.212385
Kiambu	0.217434	0.029663	0.162611	0.216219	0.279195	0.213865
Kilifi	0.197055	0.027297	0.147046	0.195775	0.254341	0.193291
Kirinyaga	0.245119	0.042587	0.163012	0.244759	0.330296	0.244804
Kisii	0.253031	0.030477	0.196588	0.251763	0.316632	0.249278
Kisumu	0.205099	0.026127	0.155398	0.204515	0.258291	0.203517
Kitui	0.306028	0.039197	0.234563	0.304022	0.388609	0.29991
Kwale	0.21321	0.039445	0.143683	0.210225	0.299837	0.204901
Laikipia	0.171268	0.030921	0.113254	0.170411	0.234597	0.16906
Lamu	0.221197	0.043751	0.140408	0.219204	0.314619	0.216482
Machakos	0.201174	0.031385	0.141462	0.200567	0.264721	0.199669
Makueni	0.354888	0.04675	0.272056	0.351717	0.454386	0.344282
Mandera	0.210569	0.047828	0.125763	0.207483	0.313107	0.201517
Marsabit	0.260956	0.05841	0.157974	0.256924	0.387094	0.249174
Meru	0.25728	0.033192	0.197082	0.255481	0.32746	0.25182
Migori	0.239612	0.035883	0.171399	0.238723	0.313353	0.237376
Mombasa	0.134289	0.029381	0.084703	0.131467	0.199849	0.126024
Murang’a	0.2378	0.034924	0.173262	0.23629	0.311028	0.233485
Nairobi	0.110499	0.014691	0.083267	0.109955	0.140854	0.108896
Nakuru	0.158986	0.023426	0.115298	0.158371	0.205751	0.156488
Nandi	0.267796	0.031096	0.210128	0.266562	0.332402	0.264094
Narok	0.306546	0.045371	0.223827	0.304174	0.402599	0.299552
Nyamira	0.219622	0.038939	0.144935	0.219341	0.29684	0.219541
Nyandarua	0.225456	0.03911	0.156202	0.222556	0.311266	0.2174
Nyeri	0.236852	0.037968	0.167805	0.234868	0.317225	0.231076
Samburu	0.188975	0.046658	0.112121	0.183797	0.295789	0.17467
Siaya	0.243214	0.030533	0.187005	0.24182	0.307268	0.239087
Taita Taveta	0.170494	0.036943	0.108046	0.166754	0.254616	0.160547
Tana River	0.234252	0.045192	0.153093	0.231357	0.332332	0.226425
Tharaka	0.289095	0.046947	0.202895	0.286759	0.38878	0.282564
Trans Nzoia	0.267196	0.040025	0.198522	0.263728	0.354138	0.25548
Turkana	0.246847	0.051781	0.152972	0.244213	0.355904	0.239152
Uasin Gishu	0.211391	0.035665	0.145082	0.209966	0.286253	0.207635
Vihiga	0.241742	0.043271	0.165836	0.238133	0.338123	0.231999
Wajir	0.195857	0.045628	0.115998	0.192536	0.294879	0.186231
West Pokot	0.289016	0.049891	0.196994	0.286656	0.394918	0.28263
UGANDA						
Adjumani	0.261711	0.018604	0.226279	0.261318	0.299419	0.260574
Amolatar	0.299037	0.019231	0.261945	0.298796	0.337505	0.298335
Amuria	0.307087	0.023696	0.261659	0.30672	0.3546	0.305994
Apac	0.291861	0.017674	0.257419	0.29176	0.326863	0.291541
Arua	0.273217	0.016167	0.242131	0.272965	0.305766	0.2725
Bugiri	0.264013	0.013069	0.238596	0.263882	0.290237	0.263672
Bukwa	0.295186	0.021655	0.253803	0.294782	0.338884	0.294011
Bundibugyo	0.194048	0.015356	0.165019	0.193649	0.225368	0.192885
Bushenyi	0.23995	0.015164	0.211125	0.239614	0.270729	0.238992
Busia	0.267063	0.015134	0.237163	0.267038	0.297066	0.266995
Butaleja	0.27925	0.01565	0.248393	0.279247	0.310067	0.279213
Gulu	0.253819	0.018003	0.219523	0.253452	0.290212	0.252741
Hoima	0.269749	0.016041	0.238712	0.269565	0.301832	0.269215
Ibanda	0.280648	0.015056	0.251733	0.280374	0.311185	0.279891
Iganga	0.227126	0.010956	0.205613	0.227061	0.249023	0.226961
Isingiro	0.253023	0.017126	0.220023	0.252794	0.287321	0.252345
Jinja	0.188422	0.012742	0.16388	0.188237	0.214019	0.187899
Kaabong	0.308241	0.047924	0.218669	0.306793	0.406056	0.303847
Kabale	0.23691	0.01224	0.212758	0.236842	0.261441	0.236733
Kabarole	0.242994	0.012847	0.218147	0.242816	0.268871	0.242489
Kaberamaido	0.268981	0.017575	0.235261	0.268697	0.304326	0.268159
Kalangala	0.253757	0.021099	0.213685	0.253295	0.296472	0.25239
Kaliro	0.277446	0.015788	0.246736	0.277303	0.308995	0.277059
Kampala	0.162551	0.015517	0.133369	0.162118	0.194195	0.161262
Kamuli	0.259509	0.015751	0.229315	0.259239	0.291271	0.258752
Kamwenge	0.254662	0.012617	0.230949	0.254238	0.281006	0.253557
Kanungu	0.281785	0.016026	0.250495	0.28164	0.313936	0.281392
Kapchorwa	0.274861	0.016567	0.24298	0.2746	0.308258	0.274123
Kasese	0.22426	0.012179	0.201361	0.223877	0.249481	0.223228
Katakwi	0.298518	0.022678	0.255107	0.298139	0.344086	0.297398
Kayunga	0.234659	0.010803	0.213414	0.234596	0.256272	0.234492
Kibaale	0.250484	0.01144	0.229596	0.249902	0.275073	0.249027
Kiboga	0.231169	0.015432	0.201803	0.230838	0.26246	0.230218
Kiruhura	0.271334	0.031191	0.212546	0.270524	0.334752	0.268909
Kisoro	0.26217	0.016806	0.229476	0.262008	0.295782	0.261716
Kitgum	0.22003	0.015787	0.190111	0.219645	0.252187	0.218928
Koboko	0.229705	0.016471	0.19858	0.22925	0.263505	0.228431
Kotido	0.283531	0.03654	0.214677	0.282593	0.357745	0.280716
Kumi	0.267738	0.019183	0.231355	0.267298	0.306652	0.266455
Kyenjojo	0.264346	0.014456	0.236167	0.264236	0.29312	0.264007
Lira	0.226631	0.013492	0.200637	0.226438	0.253744	0.226081
Luweero	0.206726	0.011504	0.184666	0.206515	0.230063	0.20616
Manafwa	0.253388	0.017569	0.21943	0.253175	0.288552	0.252767
Masaka	0.20865	0.012341	0.185985	0.208083	0.23479	0.207156
Masindi	0.244687	0.011064	0.223487	0.244456	0.267348	0.244102
Mayuge	0.273957	0.016219	0.242588	0.273762	0.306435	0.2734
Mbale	0.211104	0.011682	0.188668	0.210883	0.234875	0.210526
Mbarara	0.201694	0.012857	0.17704	0.201461	0.227688	0.201027
Mityana	0.229304	0.012048	0.20614	0.229092	0.253719	0.228723
Moroto	0.307229	0.054478	0.206489	0.305243	0.419225	0.301144
Moyo	0.215573	0.013514	0.189608	0.215284	0.243298	0.214817
Mpigi	0.247438	0.018956	0.211263	0.247077	0.285648	0.246365
Mubende	0.255686	0.016667	0.223737	0.255408	0.289215	0.254876
Mukono	0.207919	0.012186	0.183983	0.207887	0.231992	0.207796
Nakapiripirit	0.272908	0.031365	0.213563	0.272172	0.336454	0.270715
Nakaseke	0.245713	0.019944	0.207761	0.245294	0.28605	0.244472
Nakasongola	0.202663	0.012157	0.179822	0.202286	0.227814	0.201672
Nebbi	0.250149	0.01416	0.22304	0.249878	0.278854	0.249396
Ntungamo	0.267472	0.014509	0.239167	0.267362	0.296397	0.26715
Pader	0.304523	0.020966	0.264351	0.304178	0.346668	0.303518
Pallisa	0.266325	0.01506	0.236828	0.26627	0.296103	0.26614
Rakai	0.25032	0.017994	0.215877	0.250011	0.286528	0.249404
Rukungiri	0.222322	0.012229	0.198368	0.222209	0.246936	0.222015
Sembabule	0.243954	0.022513	0.201612	0.243324	0.289883	0.242081
Sironko	0.23295	0.01109	0.21204	0.232581	0.256248	0.232057
Soroti	0.298727	0.020501	0.259522	0.298367	0.340018	0.297685
Tororo	0.2622	0.016811	0.230186	0.261844	0.296288	0.261195
Wakiso	0.174291	0.013663	0.148291	0.17401	0.201898	0.173463
Yumbe	0.302381	0.020633	0.262827	0.302035	0.343916	0.301379
